# The Use of Close Friends on Instagram, Help-Seeking Willingness, and Suicidality Among Hong Kong Youth: Exploratory Sequential Mixed Methods Study

**DOI:** 10.2196/37695

**Published:** 2022-10-12

**Authors:** Sikky Shiqi Chen, Tai Pong Lam, Kwok Fai Lam, Tak Lam Lo, David Vai Kiong Chao, Ki Yan Mak, Edmund Wing Wo Lam, Wai Sin Tang, Hoi Yan Chan, Paul Siu Fai Yip

**Affiliations:** 1 Department of Family Medicine and Primary Care The University of Hong Kong Hong Kong China; 2 Department of Statistics and Actuarial Science The University of Hong Kong Hong Kong China; 3 Centre for Quantitative Medicine Duke-NUS Medical School Singapore Singapore; 4 Pamela Youde Nethersole Eastern Hospital Hong Kong China; 5 Department of Family Medicine and Primary Health Care Hospital Authority Kowloon East Cluster Hong Kong China; 6 Hong Kong Jockey Club Centre for Suicide Research and Prevention The University of Hong Kong Hong Kong China; 7 Department of Social Work and Social Administration The University of Hong Kong Hong Kong China

**Keywords:** Close Friends, private online expression, help-seeking willingness, suicide, youth

## Abstract

**Background:**

Social networking sites (SNSs) have gained popularity in recent years for help seeking and self-distress expression among adolescents. Although online suicidal expression is believed to have major benefits, various concerns have also been raised, particularly around privacy issues. Understanding youths’ help-seeking behavior on SNSs is critical for effective suicide prevention; however, most research neglects the impacts of the private SNS context.

**Objective:**

This study aims to examine youths’ private SNS use via the new Instagram feature, *Close Friends*, and its association with both online and offline help-seeking willingness as well as youths’ suicidality.

**Methods:**

This study employed an exploratory sequential mixed methods approach with a combination of explorative qualitative interviews and a systematic quantitative survey, targeting youth aged 15-19 years in Hong Kong. The motivations for utilizing Close Friends and concerns regarding online expression were addressed in the focus group and individual interviews (n=40). A cross-sectional survey (n=1676) was conducted subsequently with eligible secondary school students to examine the prevalence of Close Friends usage, their online and offline help-seeking willingness, and suicide-related experiences.

**Results:**

A total of 3 primary motives for using Close Friends were identified during interviews, including (1) interaction and help seeking, (2) release of negative emotions, and (3) ventilation and self-expression. Most participants also highlighted the privacy concerns associated with public online communication and the importance of contacting close friends for emotional support. Survey results showed that use of Close Friends was quite prevalent among adolescents (1163/1646, 70.66%), with around 46% (754/1646, 45.81%) of respondents being frequent users. Differences by gender and school academic banding were also revealed. Regarding help-seeking intentions, youths were generally positive about seeking help from peers and friends offline (1010/1266, 79.78%) yet negative about seeking assistance from online friends or professionals with whom they had not yet developed a real-world connection (173/1266, 13.67%). Most notably, frequencies of Close Friends usage were differentially associated with online and offline help-seeking willingness and youths’ suicidality. Compared with nonusers, those who had ever used the feature were more likely to seek offline support (adjusted odds ratios [AORs] 1.82-2.36), whereas heavy use of Close Friends was associated with increased odds of online help-seeking willingness (AOR 1.76, 95% CI 1.06-2.93) and a higher risk of suicidality (AOR 1.53, 95% CI 1.01-2.31).

**Conclusions:**

The popularity of Close Friends reflects the increasing need for private online expression among youth. This study demonstrates the importance of Close Friends for self-expression and private conversation and inadequacy of peer support for suicidal adolescents. Further research is needed to identify the causal relationship between Close Friends usage and help-seeking willingness to guide the advancement of suicide prevention strategies. Researchers and social media platforms may cooperate to co-design a risk monitoring system tailored to the private SNS context, assisting professionals in identifying youth at risk of suicide.

## Introduction

### Youths’ Online Expression

Online social networking sites (SNSs) are gaining increasing popularity among adolescents in recent years, changing the nature of communication. Instagram is one of the most popular SNSs worldwide [1] and the third most popular SNS in Hong Kong among people aged 16-64 years [2]. It has roughly 1 billion monthly active users [1], with around 40% of them aged 13-24 [3]. Prior studies have shown that SNSs, especially Instagram, have become a favored alternative for at-risk youth to seek help and express their distress [4,5]. Indeed, online expression pertinent to suicidality is thought to have several major benefits, including mitigated social isolation, recovery-oriented encouragement, and alleviation of acute self-harm urges [6,7]. However, in light of concerns around privacy issues, unsympathetic responses from anonymous viewers [8], and the “positivity bias,” negative disclosure on SNS is often deemed risky and thus less commonly shared publicly [9]. For example, previous research on Facebook demonstrated that most negative expressions were published exclusively on private pages as opposed to publicly [10].

To date, there has been only minimal discussion about private SNS usage among users with varying levels of mental health issues. It, therefore, remains uncertain as to how private and public online expressions differ in terms of social support seeking. This is the first study to adopt an exploratory sequential mixed methods approach to assess how private online expression is correlated with help-seeking willingness and suicidality among adolescents via *Close Friends* posts on Instagram. It was anticipated that our findings would inform the development of more effective and pragmatic suicide prevention and intervention programs delivered via SNS.

### Close Friends: A New Feature for Private Online Expression

Private online interaction has gradually developed with the advancement of technology and now varies in terms of intimacy and extent of self-disclosure [11]. Private expression on SNSs, also known as active private SNS use, is characterized by direct users’ interactions in a private setting, generally via instant messaging and personal chatting [12]. To compare the 2 modes of SNS use, public online communication allows large-scale interactions between individuals who have never met in person, whereas private online communication usually occurs in a smaller group and involves friends who have established trust and fundamental mutual understanding offline [13,14].

Aversion to publishing unpleasant content, especially mental health–related disclosures, has been observed on Instagram. On account of context collapse, the most sensitive (such as families and romantic partners) and unintended audiences can browse youths’ SNS profiles and navigate their posts [15,16]. Adolescents have shown concerns that posting depressive messages in the public space will be criticized as being offensive by the broad, diverse, or unknown audience [17]. Therefore, some create a fake Instagram account (dubbed Finsta) specifically for negative online expression [18]. On Finsta, users only follow their intimate friends so they are less worried about revealing negativity and vulnerability, and are instead prompted to present authentic and unfiltered self-expression [19]. The emerging demand for private online expression to discuss sensitive issues is reflected in the trend of multiple accounts. The traditional features of SNSs, by contrast, are deficient in terms of private expression, mostly through dyadic messages [20], making it difficult for adolescents to engage with multiple users simultaneously. The format of text-based communication is also restricted in conveying emotions precisely.

Hence, Instagram launched a new feature named Close Friends in late 2018 [21]. Only those added to the list are permitted to view private *Stories* posted in Close Friends. Followers will also be notified that they have been included in one’s Close Friends list on Instagram when they see their posts with the special icon. Compared with traditional private communication, Close Friends provides more opportunities for online expression by virtue of its unique features [22], which include the following:

- Close Friends enables parallel multiple interactions in a private context.
- Users have their own absolute control over the members’ list, which means they are able to block or remove anyone on the list at any time without notifying them.
- Posts in Close Friends do not predetermine any specific audience.
- Users are able to seek attention via posts with sensitive information, thus help seeking requires less proactivity.
- Users’ posts in Close Friends are actively selected and “pressed” to view by the audience who care about their lives.

The aforementioned properties of Close Friends distinguish its content display from those of the conventional tools for private online communication. Indeed, Close Friends is a more visually, graphically, and multimedia-oriented feature compared with instant messaging, which largely relies on text- or voice-based communications. Users of Close Friends are also able to publish posts in vivid formats, such as photos and videos, to capture, share, and showcase personal life moments [23]. Furthermore, the text-based postings in Close Friends are more aesthetically appealing than those in messages or feeds due to the availability of various fonts, animated graphics interchange format, stickers, and predesigned layouts.

### Private Online Expression, Help-Seeking Willingness, and Suicidality

Suicide is a major public health concern worldwide, particularly among youth. According to the World Health Organization, suicide is the fourth leading cause of death among those aged 15-19 [24,25]. In Hong Kong, youth aged between 15 and 24 had a suicidal rate below 10 per 100,000 [26-28], which is lower than that of their counterparts, including in European countries and most East Asian neighbors [29]. However, a rising trend in the overall suicide rate among Hong Kong adolescents has been noted throughout the last 2 decades, with rates increasing from 7.7 per 100,000 in 2000 to 9.5 per 100,000 in 2018 [30].

According to the Uses and Gratifications Theory, adolescents are proactive and goal-oriented SNS users who consider their interests and expectations while choosing and using platforms [31]. The need for satisfying diverse motives would result in different online behaviors. Hence, it is essential to acquire a comprehensive understanding of youths’ purposes of using the internet. Previous research has investigated the motivations for adolescents’ use of Instagram. One such study identified 5 motives, including social engagement, archiving, self-expression, escapism, and peeking [32], while another study recognized 4 major purposes, comprising surveillance, documentation, coolness, and creativity [33]. In addition, our earlier study examined the principal motivations of online expression among Hong Kong youth, including self-expression, emotional ventilation, life sharing and documentation, social interaction, attention seeking, and help seeking [34]. We also identified a positive association between willingness of online help seeking and the motivations of expressing emotions and opinions.

The significance of professional and nonprofessional support has been validated across different populations [35,36], and therefore, most suicide interventions encourage people to seek help. Nevertheless, evidence from previous research suggested mixed findings when it came to the relationship between suicidality and online help seeking among adolescents [37]. For example, several studies found that youth who sought help online were more likely to use the internet for suicide-related purposes [38] and experience social anxiety, psychological distress, self-harm behaviors, and suicide [39,40], while other studies reported that online communication might provide social and emotional support, which could facilitate ones’ coping with depression and stress [41-43]. In general, as a large number of studies have indicated, seeking help from peers and friends in real life is preferred by the young population, compared with formal help sources (ie, professionals) and unfamiliar people online [5,44-46]. Adolescents at lower risk of suicide and mental health problems are more likely to engage in offline help seeking from peers and friends [43,47]. Among those who prefer seeking help online, one of the main motivations is to compensate for any deficits in offline support [48,49].

To date, little attention has been paid to private SNS disclosure, particularly among adolescents. Indeed, much uncertainty still exists regarding the relationship between private online expression and suicidality. Only one very recent study of university students found that active private SNS usage was associated with a lower level of suicidality [50], whereas the frequency for each type of SNS usage was not explored. Results of a longitudinal study revealed that both heavy and suicide-related internet use were strongly associated with suicidal ideation (SI) and behaviors [51]. The subgroup with high SNS usage reported more psychiatric problems and social dysfunction as well as limited family or friend support. In terms of private SNS usage and help-seeking intentions, despite a positive association between active private Facebook usage and perceived online social support demonstrated in a youth sample [12], to our knowledge, no study has directly assessed the effect of private online expression on willingness of help seeking, either online or offline.

### Rationale for This Study

At present, very little is known about the role Close Friends plays in both online expression and help seeking for suicidality. This study is part of a larger project examining youth suicide in Hong Kong with the specific aim of exploring the use of Close Friends among adolescents. An exploratory sequential mixed methods design was adopted. Qualitative data were first collected through interviews, and major themes were generated and used to facilitate the development of the quantitative instrument. The study objectives are as follows: (1) to determine the prevalence of Close Friends among youth; (2) to ascertain the frequency, purposes, and reasons for using Close Friends; and (3) to investigate its relationship with willingness to seek help both online (from friends and professionals) and offline (from peers and friends); and (4) to examine the association between the use of Close Friends and suicidality.

## Methods

### Qualitative Approach

#### Overview

Because of the lack of prior research on Close Friends, qualitative interviews enabled us to obtain a better grasp of how it was used and viewed by youth. Considering the sensitivity of suicide-related topics, most participants first engaged in 1 of the 6 focus group discussions (3-7 persons per session), with those in each group being acquainted with each other. We then conducted 12 in-depth semistructured individual interviews for those who were unable to join focus groups or who had difficulties elaborating their stories in group interviews on account of the setting or time constraints.

#### Participant Recruitment

In total, 40 participants aged 15-18 (mean 16.3, SD 1.1) years were recruited, including 31 focus group participants (12 males and 19 females) and 12 individual interview participants (4 males and 8 females); 3 female participants took part in both an individual interview and a focus group discussion. Of the 40 participants, 35 had lived experience of suicide (ie, incidence of SI, self-harm, suicide attempts [SAs], or having helped someone in crisis). The target group for interviews was Hong Kong youth aged 15-19 years with emotional distress or suicidal concern; additionally, those with comparatively fewer problems, but who showed willingness to discuss the topic of suicide, were invited. Purposive sampling was adopted to maximize variation and assure diversity of participants’ sociodemographic characteristics (eg, social backgrounds: secondary school students, university freshmen, and school dropouts), clinical and mental health status, and suicide-related experiences. Adolescents who satisfied the recruitment criteria were asked if they were willing to attend the interviews. A major barrier to recruitment was building a rapport with youth with suicidality and earning their trust, as most tended to conceal their past out of fear of the stigma associated with suicide. To facilitate the recruitment process, we solicited recommendations from people who served, or who were closely bonded with this specific group of adolescents, including teachers, medical practitioners, and school social workers, to recommend suitable participants. The circumstances of vulnerable individuals with SA experiences, or prior diagnosis of mental illnesses, were evaluated by 2 mental health professionals to affirm their eligibility for the interviews. Invitation letters and consent forms were sent to eligible participants or, if they were under the age of 18 years, to their parents and guardians.

#### Procedure and Analysis

Considering the sensitive nature of the suicide topic, interviews were conducted in a face-to-face manner and in a natural, private, and secure environment. Open-ended questions regarding the experiences and motivations of using Close Friends, concerns about online expression, and willingness of help seeking were addressed in the interviews. All the interviews were conducted between September 2018 and November 2019. Each focus group lasted for 1-1.5 hours, and each individual interview lasted around 1 hour. Two experienced facilitators led the interviews in Cantonese (the local dialect) and took charge of data analysis to ensure data trustworthiness.

Reflexive thematic analysis was performed using an inductive approach [52,53]. We focused on both semantic and latent meanings, and adhered to the 6-step framework (Textbox 1 [54]) outlined by Braun and Clarke [54].

**The 6-step framework outlined by Braun and Clarke.**

**1. Familiarization**

- All interviews were audio recorded and transcribed verbatim. Two coders (SSC and HYC) listened to the recordings carefully, read the transcripts iteratively, shared first impressions, and took brief notes.

**2. Generating codes**

- The cleaned transcripts were entered into the NVivo database (version 12; QSR International Pty Ltd). Initially, both coders independently coded the data. Relevant, informative, and potentially interesting items were encoded with concise and clear codes. SSC and HYC then contrasted the 2 sets of codes and examined how different codes could work together across the data set.

**3. Generating initial themes**

- Upon coding completion, clustering or splitting of the valid codes was determined based on their patterns, and overarching themes were generated.

**4. Developing and revising themes**

- All the initial themes were further developed. Through team discussion with 2 senior qualitative researchers (TPL and WST), SSC and HYC revised the overlaps and divergences identified in the initial themes. SSC assessed the coherence of codes within each candidate theme and reread the transcripts to evaluate the congruence between themes and the overall data set.

**5. Refining, defining, and naming final themes**

- Feedback from the team discussion guided theme refinement. Some candidate themes were combined, split, or discarded before SSC decided and named the final themes. Related quotes under each final theme were collated.

**6. Reporting**

- The qualitative findings were reported by the research team and critically reviewed by all authors. Principal themes and subthemes were converted into the questionnaire items of the quantitative survey.

### Quantitative Approach

#### Design

A cross-sectional design was adopted in the quantitative phase by the implementation of a self-administered questionnaire survey among secondary students in Hong Kong (aged 15-19 years).

#### Sample

The target population consisted of students in grades 10-12 who were aged 15-19 years. Invitation letters were sent via postal mail to all the secondary schools in Hong Kong, with 9 schools agreeing to participate. According to the official data released by the Education Bureau of the government in 2019, 150,720 grade 10-12 students were enrolled in 504 local and international secondary schools [55]. According to epidemiological statistics, the prevalence of SI among Hong Kong youth was no more than 25% [56]. On this premise, the required sample size was calculated. Responses from 1801 respondents were expected to have a maximum estimation error (absolute precision) of *d*=0.02 from the true prevalence rate with a 95% CI.

#### Data Collection and Questionnaire

Quantitative data were collected between September and October 2019. The questionnaire was anonymous and coded with a reference number (eg, A001) to indicate the schools for data analyses. As required by the ethics committee, we developed a risk management protocol with careful consideration of both the preservation of confidentiality and the facilitation of risk control in schools. Teachers or coordinators at participating schools would be informed of the distribution of suicidal risks among their students. An alert would also be sent out to the school if a certain portion of high-risk cases (ie, ≥25%; referring to youth suicidality rate in Hong Kong) were identified [56].

Questionnaire items were stemmed from qualitative findings, a review of the literature, and feedback from the research team. The final survey consisted of 51 items and took around 15-20 minutes to complete. As part of the larger project, this study contained several sections of the questionnaire, including the frequency (measured on a 4-point Likert scale: 1, never; 2, seldom; 3, sometimes; and 4, often; response options are comparable with those adopted in a previous study [50]) of using Close Friends, willingness of online help seeking, willingness of seeking help from peers and friends, SI and SA experiences in the past 12 months, and sociodemographic information.

To measure respondents’ help-seeking willingness, we posed the following question: “When confronted with distressing issues or life difficulties, did you seek help from any of the following in the last 12 months?” The response options were dichotomous (coded as *Yes*=1 or *No*=0) and included both online (online friends and online professionals) and offline (peers, friends, and classmates) resources of help. Respondents’ suicide risk was examined by questions on prior suicidal behaviors scored as *Yes*=1 or *No*=0. We asked respondents “Have you considered killing yourself in the past 12 months” and “Have you attempted to kill yourself during the past 12 months?” Nonaffirmative responses to both questions were categorized as “no/low risk” of suicide, affirmative responses to the first question only as “medium risk,” and affirmative responses to both questions as “high risk.” Investigation on recent SI and behaviors was conducted to compare the relative levels of suicidality in the population. Most items were either binary or categorical. The questionnaire was pilot-tested for its reliability and validity. After minor modifications were made, the questionnaires were then distributed to all the eligible students in the participating schools.

#### Statistical Analysis

The quantitative data were analyzed using SPSS (version 27.0; IBM Corp). Data cleansing followed the recommendations on treating univariate and multivariate outliers [57]. The results of missing values analysis indicated satisfaction on criteria (Little test) for missing completely at random (*χ*^2^_1_=2.3; *P*=.13), and missing values were replaced through the expectation maximization method. To summarize the distribution of responses on each item, descriptive statistics were presented by frequencies and percentages. Sensitivity analyses using the Pearson chi-square test were performed to examine whether differences in Close Friends use and help-seeking willingness were attributable to respondents’ backgrounds or suicide-related experiences. We also used ordinal logistic regression to estimate odds ratios (ORs), adjusted ORs (AORs), and 95% CIs for the associations of the frequency of Close Friends usage with willingness of help seeking and suicidality. Statistical significance was indicated with a *P* value <.05.

### Ethics Approval

Ethical approval was obtained from the local Institutional Review Board of The University of Hong Kong/Hospital Authority Hong Kong West Cluster (approval number UW 18-338).

## Results

### Qualitative Findings

#### Use of Close Friends and Themes Identified

The usage of Close Friends was mentioned in half of the individual interviews, and 2 out of 6 focus groups addressed the concern regarding public online expression and emotional sharing. Participants who had no experience with private online expression indicated a preference for seeking emotional support from close friends.

The following 3 recurrent themes emerged from the analysis with regard to the usage of Close Friends: (1) reasons for using Close Friends, (2) general concerns about privacy issues, and (3) the importance of seeking help from Close Friends for emotional problems. Each theme is discussed in detail in the next section and the narrative is supported by the illustration of pertinent quotes. Study participants are identified by the interview type and number, gender (“M” and “F” refer to males and females, respectively), age, and suicide-related experiences (“with SI/no SI” indicating whether they had any SI; attempter/nonattempter indicates SA experiences).

#### Reasons for Using Close Friends: Results From Individual Interviews

##### Overview

The 3 main reasons for using Close Friends were identified from the individual interviews. These reasons included (1) interaction and help seeking, (2) negative emotions release, and (3) ventilation and self-expression. Relevant quotes are selected and illustrated in the following sections.

##### Interaction and Help Seeking

Close Friends was commonly used by participants to “interact and seek help from friends”. Because of the great heterogeneity of viewers, some participants had difficulty getting support via public posts, while others were hesitant to disturb friends via direct messaging. Therefore, posting on Close Friends turned out to be an ideal option for sharing problems or challenges. It could be an advance notice for friends in real life that a follow-up discussion on the issue was anticipated when they met face-to-face the following day.

I never post public stories but always use Close Friends when I post stories on Instagram. There are many people I don’t know on this platform. I don’t want them to know how I feel. Also, they may not know me, so it is meaningless for them to see the post. Sometimes I can’t tell my friends right away after arguing with my dad at night, so I would post [to Close Friends] on Instagram. Or maybe there is nothing serious, and I just want to mention it to them. We can discuss it face to face directly the next day.

Individual-12, M, 15 years, secondary school student, no SI, nonattempter

##### Negative Emotions Release

Close Friends is also a haven for those who are determined to build a favorable public image on social media as it provides a private space for them to “release negative emotions.” Indeed, several participants were concerned about how their posts might be treated and whether their posts with emotional expression would demote their prestige, particularly in the eyes of strangers. Thus, using Close Friends made it psychologically safer to publish unfavorable information on SNSs.

I prefer to post photos with individualized characteristics to bring a positive feeling to others, and I won’t post any negative stuff on social media. However, thanks to Instagram Stories and a new feature called Close Friends, I share more about my daily life [as well as negative emotions]. After all, Instagram is a popular platform with a large number of targeted audiences. I don’t want people to think I’m too negative [so I won’t post negative things in public].

Individual-5, F, 18 years, university freshman, with SI, nonattempter

##### Ventilation and Self-expression

In addition, as Close Friends posts can only be viewed and commented on by a limited number of followers, some participants believed Close Friends was a suitable outlet to “ventilate” and facilitated their willingness of online expression.

I have seen someone put the image of wrist cutting on the Internet, but I would not do the same thing. I would regard [posting online] as one of the ways to ventilate, and I [tended to] say things in a tactful and restrained manner. I use Instagram, but I only have a few followers. Most of them are my close friends. Sometimes I don't think they could understand me, so I will treat the posts as if I am speaking to myself.

Individual-4, F, 16 years, secondary school student, with SI, attempter

#### General Concerns of Privacy Issues: Results From Focus Groups

However, none of the participants from the focus group interviews mentioned their experiences of using Close Friends, although a few did address the privacy concerns when expressing emotions on social media.

There was no way to ventilate before, because online platforms were poorly developed then. But now, even if [online platforms are much better developed comparatively], when you have something to share, something you don’t like, or you are uncomfortable with, you would choose a group of familiar close friends [instead of everyone online], that is, you will share it in a small circle.

Group 5: Participant-4, M, 18 years, secondary school student, no SI, nonattempter

IG (Instagram) posts may be viewed by too many people, so I won’t post [my status] on it. I will talk to close friends [if I have something to share] by WhatsApp message.

Group 6: Participant-4, F, 16 years, secondary school student, no SI, nonattempter

#### Help Seeking: Importance of Contacting Close Friends for Emotional Problems

Close Friends is a relatively new feature on Instagram; consequently, some participants might not have known about it at the time of the interviews. This could explain why some participants highlighted the importance of contacting and seeking help from close friends but had never actually used Close Friends themselves. It is therefore possible that Close Friends might change their attitudes toward online expression and that these participants might have a greater interest in posting on Instagram after learning about the function.

You should find some close friends to chat with, but not with some people who don't know you entirely. A close friend means someone who knows your personality and your ways of doing things. Those who don't know you may only be able to give some poor suggestions.

Individual-1, F, 16 years, school dropout, with SI, nonattempter

I would talk to my friends about my personal matters in private and I rarely posted the whole story on my Instagram account. I usually shared it in the WhatsApp group since I didn’t dare...[directly posting it in public]. I wanted to find someone to listen to me, but I don’t like being judged.

Individual-11, M, 15 years, secondary school student, with SI, nonattempter

### Results of the Questionnaire Survey

#### Respondents to Questionnaires

Of the 1704 returned questionnaires, 1676 contained valid responses, including 822/1658 from males (49.58%) and 836/1658 from females (50.42%) with a mean age of 16.0 (SD 1.2) years. The number of students in each of the 3 tiers of school bandings was distributed evenly. Bandings are assigned to schools based on students’ academic performance in ascending order. Generally speaking, students attending Band 1 schools scored higher on the university entrance examination.

**Figure 1:**
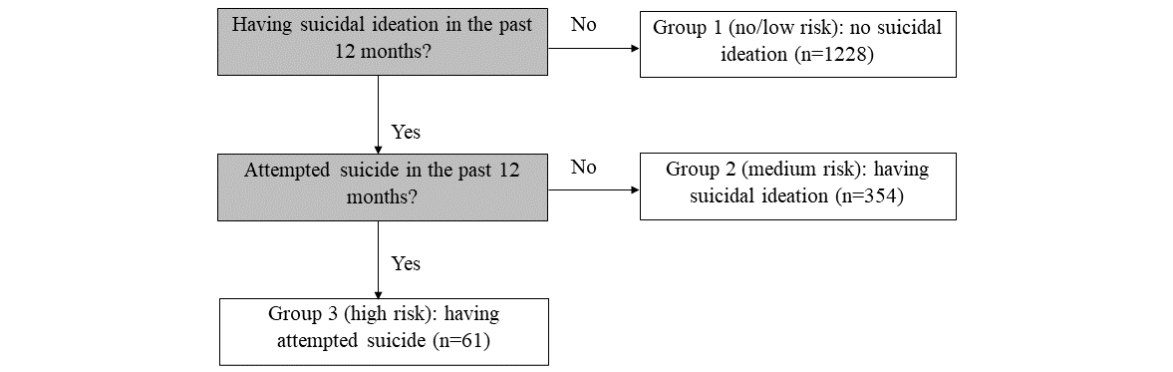
Categorization of the 3 groups for suicidality based on indicated suicide-related experiences.

With regard to suicidality, the following 3 groups were identified based on indicated SI and SA experiences over the last 12 months: (1) 1228/1643 (74.74%) respondents reported that they had no SI; (2) 354/1643 (21.55%) respondents reported that they only had SI; and (3) 61/1643 (3.71%) respondents reported that they had attempted suicide. Accordingly, respondents’ suicidality was categorized into 3 groups, including “no/low risk” (no SI), “medium risk” (with SI only), and “high risk” (with SA). The procedure of categorization of respondents’ suicidality is depicted in Figure 1.

#### Frequency of Using Close Friends and Respondents’ Help-Seeking Willingness

More than 70% (1163/1646, 70.66%) of the respondents had ever used Close Friends and around 46% (754/1646, 45.81%; 95% CI 0.43-0.48) were frequent users (those who selected “sometimes” and “often”). Around 80% (1010/1266, 79.78%) of the respondents were willing to seek help from peers and friends, yet less than 15% (173/1266, 13.67%) of them indicated an interest in seeking help online for self-distress. The details of respondents’ sociodemographic characteristics, help-seeking willingness, and usage of Close Friends are presented in Table 1.

**Table 1:** Respondents’ sociodemographic characteristics, willingness of help seeking, and frequency of using Close Friends (n=1676).

Sociodemographic characteristics				Value, n (%)
**Gender (n=1658)^a^**				
	Male			822 (49.58)
	Female			836 (50.42)
**School banding**				
	Band 1			442 (26.37)
	Band 2			578 (34.49)
	Band 3			656 (39.14)
**Willingness of help seeking (n=1266)^b^**				
	**Online**			
		Yes	173 (13.67)	
	**From peers and friends**			
		Yes	1010 (79.78)	
**Frequency of using Close Friends (n=1646)^c^**				
	Never			483 (29.34)
	Seldom			409 (24.85)
	Sometimes			492 (29.89)
	Often			262 (15.92)

^a^In total, 18 respondents failed to report their genders. These missing values were acceptable considering the size of the entire data set. The valid percentages were thus calculated by 1658 responses.

^b^The valid percentages of willingness to seek help online or from peers and friends were calculated among those who reported willingness to seek help (n=1266).

^c^There were 30 respondents who failed to report the frequency of using Close Friends. These missing values were acceptable considering the size of the entire data set. The valid percentages were thus calculated by 1646 responses.

#### Differences in the Frequency of Using Close Friends and Help-Seeking Willingness by Respondents’ Background Characteristics

Table 2 presents the differences in the frequency of using Close Friends and willingness of help seeking among respondents with varying sociodemographic characteristics and suicide-related experiences. It was found that around 40% (295/802, 36.8%) of male respondents had never used Close Friends, while over half of the female respondents were frequent users (those who answered “sometimes” and “often”) of Close Friends (*χ*^2^_3_=60.2; *P*<.001). Band 1 school students more often used Close Friends (*χ*^2^_6_=12.9; *P*=.04). With regard to help-seeking willingness, females were more active in both online help seeking (*χ*^2^_2_=5.8; *P*=.02) and peer and friend–based help seeking (*χ*^2^_1_=15.3; *P*<.001). Students from Band 1 schools were less likely to seek help online (*χ*^2^_2_=9.3; *P*=.009). Respondents who were willing to seek help from peers and friends were less likely to develop SI (*χ*^2^_2_=11.8; *P*=.003), whereas those who were willing to seek help online were more likely to have SI and SA experiences (*χ*^2^_2_=9.0; *P*=.01).

**Table 2:** Willingness of help seeking and the frequency of using Close Friends by respondents’ background characteristics (n=1676).^a^

Variables		Gender										School bandings											Suicide-related experiences^b^								
		Male, n/N (%)			Female, n/N (%)		Chi-square (*df*)		*P* value		Band 1, n/N (%)			Band 2, n/N (%)		Band 3, n/N (%)		Chi-square (*df*)		*P* value		Group 1, n/N (%)			Group 2, n/N (%)		Group 3, n/N (%)		Chi-square (*df*)		*P* value
Online help-seeking willingness		67/597 (11.2)			105/660 (15.9)		5.8 (1)		.02		32/345 (9.3)			71/421 (16.9)		70/501 (14.0)		9.3 (2)		.009		117/964 (12.1)			48/247 (19.4)		6/39 (15.4)		9.0 (2)		.01
Peer-oriented help-seeking willingness		450/597 (75.4)			555/659 (84.1)		15.3 (1)		<.001		278/345 (80.6)			340/420 (81.0)		392/501 (78.2)		1.2 (2)		.54		788/963 (81.8)			178/247 (72.1)		30/39 (76.9)		11.8 (2)		.003
**Frequency of Close Friends use**							60.2 (3)		<.001									12.9 (6)		.04									6.4 (6)		.38
	Never		295/802 (36.8)	181/826 (21.9)						110/437 (25.2)			182/574 (31.7)		191/635 (30.1)						349/1205 (29.0)			100/349 (28.7)		20/60 (33.3)					
	Seldom		210/802 (26.2)	194/826 (23.5)						97/437 (22.2)			145/574 (25.3)		167/635 (26.3)						315/1205 (26.1)			77/349 (22.1)		12/60 (20.0)					
	Sometimes		200/802 (24.9)	288/826 (34.9)						144/437 (33.0)			162/574 (28.2)		186/635 (29.3)						362/1205 (30.0)			110/349 (31.5)		15/60 (25.0)					
	Often		97/802 (12.1)	163/826 (19.7)						86/437 (19.7)			85/574 (14.8)		91/635 (14.3)						179/1205 (14.9)			62/349 (17.8)		13/60 (21.7)					

^a^Pearson chi-square tests were used to analyze the data.

^b^The 3 groups were divided for suicidality based on reported suicide-related experiences: group 1 includes those who had no SI, group 2 includes those had SI, and group 3 includes those who had attempted suicide.

#### Association of the Frequency of Using Close Friends With Help-Seeking Willingness

Table 3 presents the association of the frequency of using Close Friends with online and peer-oriented help-seeking willingness. After adjustment for gender and school banding effect, those who had ever used Close Friends were significantly more (*P*<.001 for “often”, *P*<.001 for “sometimes”, and *P*=.001 for “seldom”) likely to seek help from peers and friends than those who had never used the feature (AORs 1.82-3.02), independent of the frequency with which the feature was used. Respondents who posted in Close Friends most frequently (response of “often”) had a higher likelihood (*P*=.03) to seek help online when compared with those who never used the feature (AOR 1.76, 95% CI 1.06-2.93). However, no significant association has been found between the other 2 levels of usage frequency (responses of “sometimes” and “seldom”) and online help-seeking willingness (*P*=.44 for “sometimes” and *P*=.16 for “seldom”).

**Table 3:** Summary of ordinal logistic regression analyses for the association of help-seeking willingness with the frequency of using Close Friends (n=1676).^a^

Predictor: Frequency of Close Friends use^b^			Model 1									Model 2^c^						
			Estimate		SE		*P* value		Crude odds ratio (95% CI)		Estimate			SE		*P* value		Adjusted odds ratio (95% CI)
**Outcome 1: Willingness of online help seeking** ^d^																		
	Frequency: often	0.62		0.25		.02		1.85 (1.13-3.04)		0.57			0.26		.03		1.76 (1.06-2.93)	
	Frequency: sometimes	0.23		0.24		.33		1.26 (0.79-1.99)		0.19			0.24		.44		1.20 (0.75-1.92)	
	Frequency: seldom	0.37		0.24		.13		1.44 (0.90-2.31)		0.35			0.24		.16		1.41 (0.88-2.27)	
**Outcome 2: Willingness of seeking help from peers and friends** ^e^																		
	Frequency: often	0.94		0.22		<.001		2.57 (1.65-3.98)		0.86			0.23		<.001		2.36 (1.51-3.70)	
	Frequency: sometimes	1.13		0.19		<.001		3.10 (2.13-4.51)		1.11			0.20		<.001		3.02 (2.06-4.43)	
	Frequency: seldom	0.58		0.18		.002		1.78 (1.24-2.55)		0.60			0.19		.001		1.82 (1.27-2.63)	

^a^Ordinal logistic regression analyses were used to analyze the data.

^b^For the predictor in both analyses, the frequency of using Close Friends has 4 outcome levels in ascending order: never, seldom, sometimes, and often. The response of “never” was chosen as the reference category.

^c^Model 2 adjusted for gender and school banding.

^d^For outcome 1: willingness of online help seeking, the response of “no” was chosen as the reference category.

^e^For outcome 2: willingness of seeking help from peers and friends, the response of “no” was chosen as the reference category.

#### Association of Suicidality With the Frequency of Close Friends Use and Willingness of Help Seeking

Table 4 shows the risk factors for suicidality. After adjustment for the gender and school banding effect, willingness to seek help online was associated with an increased risk of suicidality (AOR 1.50, 95% CI 1.04-2.15), while willingness to seek help from peers and friends was associated with a decreased risk of suicidality (AOR 0.55, 95% CI 0.39-0.75). In terms of Close Friends usage frequency, those who “sometimes” used Close Friends had an elevated risk of suicidality compared with those who had never used Close Friends (AOR 1.53, 95% CI 1.01-2.31).

**Table 4:** Summary of ordinal logistic regression analyses for risk of suicidality (n=1676).^a^

Outcome: risk of suicidality^b^		Model 1					Model 2^c^			
		Estimate	SE	*P* value	Crude odds ratio (95% CI)	Estimate		SE	*P* value	Adjusted odds ratio (95% CI)
**Predictor 1: Frequency of Close Friends use (Reference: never)**										
	Often	0.47	0.21	.03	1.60 (1.06-2.41)	0.40		0.22	.07	1.49 (0.98-2.28)
	Sometimes	0.44	0.21	.03	1.56 (1.04-2.34)	0.42		0.21	.047	1.53 (1.01-2.31)
	Seldom	0.19	0.19	.34	1.21 (0.82-1.76)	0.23		0.20	.25	1.26 (0.85-1.85)
**Predictor 2: Online help-seeking willingness (Reference: no)**										
	Yes	0.46	0.18	.01	1.59 (1.11-2.27)	0.40		0.19	.03	1.50 (1.04-2.15)
**Predictor 3:** **Peer-oriented help-seeking willingness (Reference: no)**										
	Yes	–0.58	0.16	<.001	0.56 (0.41-0.77)	–0.61		0.17	<.001	0.55 (0.39-0.75)

^a^Ordinal logistic regression analyses were used to analyze the data.

^b^Risk of suicidality had 3 outcome levels: “no ideation,” “having SI,” and “having attempted suicide.” The response of “no ideation” was chosen as the reference category.

^c^Model 2 adjusted for gender and school banding.

## Discussion

### Principal Findings

To the best of our knowledge, this is the first study on Close Friends, a newly introduced feature of Instagram. This is also the first rigorous evaluation to explore how the private expression features on SNSs may influence adolescents’ willingness of online expression and help seeking with regard to mental distress and suicidality. Our findings demonstrated that a sizable proportion (1163/1646, 70.66%) of adolescents had ever used Close Friends, with around half of the respondents (754/1646, 45.81%) being frequent users. We identified 3 major motivations for using Close Friends during interviews, including (1) interaction and help seeking, (2) release of negative emotions, and (3) ventilation and self-expression. In terms of help-seeking willingness, youths were largely positive toward seeking help from peers and friends offline, yet negative toward online help seeking from online professionals or online friends with whom they had not yet established a relationship in real life. Most notably, we identified a positive association between the frequency of using Close Friends and the willingness to seek help from peers and friends, as well as a tendency for those who heavily used Close Friends to be predisposed to a higher suicide risk and greater online help-seeking willingness, compared with nonusers. Willingness to seek help online was shown to be positively correlated with suicidality, whereas willingness to seek help from peers and friends was found to be negatively associated with suicidality. The findings of our study, therefore, significantly contribute to the field by highlighting the emerging trend of private online expression and its relationship with suicidality and help-seeking willingness among adolescents. Moreover, it will be effective to raise awareness of suicide prevention in the public so that we can all learn to be the guardian angels of others on social media.

### Prevalence of Close Friends Usage and Sociodemographic Variations in Their Use

Our findings show a relatively high prevalence of Close Friends usage among youth. The popularity of Close Friends is as predicted, because personal disclosure has been one of the central aspects of SNS. A higher acceptance rate was found on Instagram among adolescents for distress expression, owing to its advanced privacy settings [58]. The gender differences identified were in line with what was found in the majority of previous studies [59,60]. Interestingly, students from Band 1 schools used Close Friends more often, which contradicted the findings of most prior studies, where social media usage had little or a negative association with academic performance [61,62]. This could be attributable to the study populations (university students vs secondary school students) and intents of using SNSs (entertainment vs private conversation).

### Private Online Expression, Willingness of Help Seeking, and Suicidality

Understanding the topic of private online expression and its association with help-seeking willingness and suicidality remains in a nascent state [63]. Our findings suggest that the new feature could substantially facilitate offline help seeking. Notwithstanding, the higher-frequency use of Close Friends may promote online help seeking, whereas the risk of suicidality would increase concurrently. This finding contrasted with previous research indicating that only passive use of SNS (ie, viewing posts), but not active, was linked with a decrease in one’s subjective well-being [64,65], and suggested that the frequency of SNS usage could be the determining factor that resulted in the differences. While some studies reported that using SNSs may alleviate loneliness and enhance happiness, others remarked that excessive usage of SNS and online expression can exacerbate the sense of loneliness and impair one’s well-being [66]. Similarly, although using Close Friends for private online communication may help strengthen real-world social connections and maintain better social capital, heavy use of the feature may suggest underlying psychological or social malfunction, such as social media addiction, smartphone dependency, lack of confidence in person-to-person interaction, and low self-esteem, all of which indicate poorer mental health [67]. In addition, there is conflicting evidence on the relationship between time spent on SNSs and online help seeking for suicidality [38,40,68], implying that other purposes and motivations of using SNSs may contribute to the variations of both public and private online expression. Considering the basic binary categorization of passive and active use of SNS in most previous studies, future studies may explore the purposes of using SNSs in each individual construct embodied by multiple components.

In other respects, consistent with previous research [69,70], we identified a higher willingness of seeking help from peers and friends and a lower willingness of online help seeking. Besides the lack of knowledge and mental health literacy, based on what interview participants stressed, privacy concerns and the priority of close friends for personal emotions may account for the overall preferences of offline help seeking. Our findings on the association between suicidality and help-seeking willingness are also supported by empirical evidence. For example, previous studies revealed that peer-related loneliness was positively associated with nonsuicidal self-injury (NSSI) engagement [71] and that Chinese adolescents with online help-seeking behaviors had a greater lifetime prevalence of SI [72]. With specific regard to online help seeking, previous research indicated that youngsters with lower life satisfaction and a higher level of stress are more prone to seeking help online [73]. By contrast, although our study provides no evidence of the significant relationship between willingness of online and offline help seeking, a previous study reported that young people who had sought help online for suicide-related issues were less likely to disclose to someone offline [39]. Given that vulnerable youth often report a higher probability of being isolated by peers and alienated from social circles [74], this might explain why they have to turn to people online for help and support. The support from Close Friends could even be more crucial among this group of users.

### Implications for Future Studies

Considering the high prevalence of Close Friends among adolescents, this kind of private online expression may shape the behavioral patterns of help seeking. Close Friends provides a secure space for private communication and online expression, which encourages at-risk adolescents to be more authentic in self-disclosure of distress and identity exploration. Increased self-disclosure with intimate friends would, in turn, reciprocally enhance the quality of friendship, facilitate relationship development, and lead to stronger and more stable peer support [75]. Nonetheless, the high level of confidentiality inherent in the Close Friends feature may be challenging for online help services. Albeit previous research shed light on the efficacy of suicide prevention messaging [76] and professional-led online risk screening [72], with the use of Close Friends some negative expressions on SNSs would be circulated within sealed social circles where external access is entirely prohibited. As a consequence, external service providers would have a little chance to view the messages requesting assistance, and the private use of SNS would make it more controversial for professionals to access and gather data from individuals’ social media due to ethical and privacy concerns [77]. In addition, similar to Snapchat and discussion boards [78], Close Friends posts are less content visible and bear more information-sharing affordances. Qualitative findings demonstrate that negative emotional release and ventilation are the 2 primary motivations for youth to initiate expression on Close Friends, and having a private profile (only visible to “friends”) on Facebook has been shown to be negatively associated with social capital [79]. Therefore, adolescents who are deeply engaged in posting on Close Friends may be more exposed to peer hostility and contentious comments. Given the correlation between higher-frequency use of Close Friends and online help seeking, researchers and program administrators should take the use of Close Friends into careful consideration and propose solutions to the “blocking” situation.

This study adds evidence to the critical role of peers and friends in youths’ help seeking and suicide prevention. A study on friend SNS underscored the value of positive peer evaluation on SNSs for the social adjustment of adolescents [80]. Peer and friend support may reassure at-risk adolescents that they are understood by someone they trust, and even deter them from ongoing or subsequent suicidal behaviors. It is critical to promote or foster some good practices among Close Friends users that encourage them to be attentive to one another’s needs, and to seek help from outside if a situation within Close Friends requires immediate attention for the sake of its members’ safety. By contrast, although most adolescents intend to help peers in crisis when they read their NSSI posts, some argued that the peer support was not very useful and had little bearing on the decrease in their actual NSSI [81]. One probable explanation is the absence of further guidance in professional consultation, as adolescents often inquire about professionals’ suggestions for a peer’s condition, but rarely urge the individual to seek formal help directly [82]. Therefore, greater emphasis should be garnered on ways to improve peer training interventions and advocate for peer support services such that everyone can look after each other by providing timely mutual support.

### Limitations

This study has a number of limitations. First, the Close Friends feature was not released until midway through the focus groups, which limited how much information we were able to obtain on private expression. Considering suicide is usually a stigmatized topic, the more anonymous data collection approach (eg, online interviews and online surveys) would generate responses with greater validity compared with face-to-face interviews. Second, a validated, multi-item instrument is required in future research that evaluates suicidality and help-seeking willingness. This study included only basic measuring items and demonstrated a simple 3-level structure to indicate youths’ suicide risks. Given the mixed relationship between suicidal thoughts, past SAs, and future suicide risk, such a design inferred a certain degree of invalidity. In addition, the use of binary items greatly omitted the details regarding youths’ help-seeking willingness and reduced the validity of that information. The result of logistic rather than linear regression could only be considered “preliminary.” To expand our knowledge of the nuances of these associations, validated tools that thoroughly assess help-seeking and suicidality are needed. Third, private online expression is a new field of research with no validated questionnaire or scale currently available. Therefore, the items in our survey were derived mainly from qualitative results and the literature and should be revised and tested recurrently in future studies on a similar theme. Additional confounding factors should also be measured, including the number of close friends, relationship issues, parental and school support, and habits of using smartphones and SNSs. Moreover, the number of valid responses to the questionnaire survey fell short of the expected sample size due to an unanticipated amount of missing data and the withdrawal of some schools at the last moment.

### Conclusions

The popularity of Close Friends represents the proliferated need for private online expression, reflecting an emerging trend among young people for exchanging suicide-related information. This study demonstrates support for Close Friends usage for self-expression and private conversation among Hong Kong adolescents aged 15-19 years and indicates the relevance and insufficiency of current peer support for suicidal youth. Further studies should be conducted to determine the causal relationship between the frequency and purposes of using Close Friends and willingness to seek help, which would provide more information for the development of suicide prevention initiatives. Researchers and social media platforms should exercise caution when considering the impacts of heavy Close Friends usage and may also collaborate to co-design a risk monitoring system adapted to the private SNS context. Such a system would need to ensure that adolescents’ privacy is not jeopardized when communicating online as well as efficiently assist professionals in identifying young people at a high risk of suicide and notify them of any suicide-related information posted online.

## Abbreviations

AOR: adjusted odds ratio
NSSI: nonsuicidal self-injury
OR: odds ratio
SA: suicide attempt
SI: suicidal ideation
SNS: social networking site

## Data Availability

The data sets generated and analyzed during this study are available from the corresponding author on reasonable request.

## References

[1] (2022). Most popular social networks worldwide as of January 2022, ranked by number of monthly active users (in millions). Statista.

[2] (2022). Digital 2022: Hong Kong. Datareportal.

[3] (2022). Distribution of Instagram users worldwide as of April 2022, by age group. Statista.

[4] Biddle L, Derges J, Goldsmith C, Donovan JL, Gunnell D (2018). Using the internet for suicide-related purposes: Contrasting findings from young people in the community and self-harm patients admitted to hospital. PLoS One.

[5] Prescott J, Hanley T, Ujhelyi K (2017). Peer Communication in Online Mental Health Forums for Young People: Directional and Nondirectional Support. JMIR Ment Health.

[6] Dyson MP, Hartling L, Shulhan J, Chisholm A, Milne A, Sundar P, Scott SD, Newton AS (2016). A Systematic Review of Social Media Use to Discuss and View Deliberate Self-Harm Acts. PLoS One.

[7] Lewis SP, Seko Y (2016). A Double-Edged Sword: A Review of Benefits and Risks of Online Nonsuicidal Self-Injury Activities. J Clin Psychol.

[8] Nasier B, Gibson K, Trnka S (2021). “PM me” or “LOL”: Young peoples’ observations of supportive and unsympathetic responses to distress on social media. Computers in Human Behavior.

[9] Forest AL, Wood JV (2012). When social networking is not working: individuals with low self-esteem recognize but do not reap the benefits of self-disclosure on Facebook. Psychol Sci.

[10] Bazarova N, Taft J, Choi Y, Cosley D (2012). Managing Impressions and Relationships on Facebook. Journal of Language and Social Psychology.

[11] Valkenburg P, Peter J (2009). Social Consequences of the Internet for Adolescents. Curr Dir Psychol Sci.

[12] Frison E, Eggermont S (2015). Toward an Integrated and Differential Approach to the Relationships Between Loneliness, Different Types of Facebook Use, and Adolescents’ Depressed Mood. Communication Research.

[13] Boyd D, Ellison N (2007). Social Network Sites: Definition, History, and Scholarship. Journal of Computer-Mediated Communication.

[14] Chan G (2020). Intimacy, friendship, and forms of online communication among hidden youth in Hong Kong. Computers in Human Behavior.

[15] Agosto D, Abbas J (2016). “Don’t be dumb—that’s the rule I try to live by”: A closer look at older teens’ online privacy and safety attitudes. New Media & Society.

[16] Marwick A, Boyd D (2010). I tweet honestly, I tweet passionately: Twitter users, context collapse, and the imagined audience. New Media & Society.

[17] Budenz A, Klassen A, Purtle J, Yom-Tov E, Yudell M, Massey P (2022). "If I was to post something, it would be too vulnerable:" University students and mental health disclosures on instagram. J Am Coll Health.

[18] Sofia D, Schinria I, Elizabeth R, Niloufar S (2019). Finsta: Creating "Fake" Spaces for Authentic Performance. CHI '19: Proceedings of the 2019 CHI Conference on Human Factors in Computing Systems.

[19] Kang J, Wei L (2020). Let me be at my funniest: Instagram users’ motivations for using Finsta (a.k.a., fake Instagram). The Social Science Journal.

[20] Peter J, Valkenburg P (2016). Research Note: Individual Differences in Perceptions of Internet Communication. European Journal of Communication.

[21] Meta (2021). Instagram Stories. Instagram.

[22] Meta (2021). How do I share a story with my close friends list on Instagram?. Instagram.

[23] Sihombing L, Aninda M (2022). Phenomenology Of Using Instagram Close Friend Features For Self Disclosure Improvement. Professional: Jurnal Komunikasi dan Administrasi Publik.

[24] World Health Organization (WHO) (2021). Suicide. WHO.

[25] Yip PSF, Zheng Y, Wong C (2022). Demographic and epidemiological decomposition analysis of global changes in suicide rates and numbers over the period 1990-2019. Inj Prev.

[26] Pirkis J, Amadeo S, Beautrais A, Phillips M, Yip PSF (2020). Suicide Prevention in the Western Pacific Region. Crisis.

[27] Committee on Prevention of Student Suicides (2016). Final Report November 2016 by Committee on Prevention of Student Suicides. The Government of Hong Kong Special Administrative Region.

[28] Yang C, Yip PSF (2021). Changes in the epidemiological profile of suicide in Hong Kong: a 40-year retrospective decomposition analysis. China Popul Dev Stud.

[29] World Health Organization (WHO) (2014). Preventing suicide: A global imperative. WHO.

[30] HKJC Centre for Suicide Research and Prevention (2022). Suicide rates by age groups in Hong Kong, 2011-2021. HKJC Centre for Suicide Research and Prevention.

[31] Elmquist D, McLaughlin C (2017). Social Media Use Among Adolescents Coping with Mental Health. Contemp School Psychol.

[32] Lee E, Lee J, Moon JH, Sung Y (2015). Pictures Speak Louder than Words: Motivations for Using Instagram. Cyberpsychol Behav Soc Netw.

[33] Sheldon P, Bryant K (2016). Instagram: Motives for its use and relationship to narcissism and contextual age. Computers in Human Behavior.

[34] Chen SS, Lam TP, Lam KF, Lo TL, Chao DVK, Mak KY, Lam EWW, Tang WS, Chan HY, Yip PSF (2022). Motivations for Online Expression, Willingness of Online Help-Seeking, and the Risk of Suicide Among Hong Kong Youths: A Mixed-Methods Study. Cyberpsychol Behav Soc Netw.

[35] Robinson J, Bailey E, Witt K, Stefanac N, Milner A, Currier D, Pirkis J, Condron P, Hetrick S (2018). What Works in Youth Suicide Prevention? A Systematic Review and Meta-Analysis. EClinicalMedicine.

[36] Schlichthorst M, Ozols I, Reifels L, Morgan A (2020). Lived experience peer support programs for suicide prevention: a systematic scoping review. Int J Ment Health Syst.

[37] Kitagawa Y, Shimodera S, Togo F, Okazaki Y, Nishida A, Sasaki T (2014). Suicidal feelings interfere with help-seeking in bullied adolescents [corrected]. PLoS One.

[38] Harris KM, McLean JP, Sheffield J (2009). Examining suicide-risk individuals who go online for suicide-related purposes. Arch Suicide Res.

[39] Bell J, Mok K, Gardiner E, Pirkis J (2018). Suicide-Related Internet Use Among Suicidal Young People in the UK: Characteristics of Users, Effects of Use, and Barriers to Offline Help-Seeking. Arch Suicide Res.

[40] Harris KM, McLean JP, Sheffield J (2014). Suicidal and online: how do online behaviors inform us of this high-risk population?. Death Stud.

[41] Wright KB, Rosenberg J, Egbert N, Ploeger NA, Bernard DR, King S (2013). Communication competence, social support, and depression among college students: a model of facebook and face-to-face support network influence. J Health Commun.

[42] Wright K (2012). Emotional Support and Perceived Stress Among College Students Using Facebook.com: An Exploration of the Relationship Between Source Perceptions and Emotional Support. Communication Research Reports.

[43] Wong K, Chan CS, Chan M, Wong C, Cheng Q, Xiong C, Yip P (2021). Who seeks help online? Comparing online and offline help-seeking preferences amongst youths with suicidal ideation. J Affect Disord.

[44] Lim S, Chan Y, Vadrevu S, Basnyat I (2013). Managing peer relationships online – Investigating the use of Facebook by juvenile delinquents and youths-at-risk. Computers in Human Behavior.

[45] Cui S, Cheng Y, Xu Z, Chen D, Wang Y (2011). Peer relationships and suicide ideation and attempts among Chinese adolescents. Child Care Health Dev.

[46] D'Avanzo B, Barbato A, Erzegovesi S, Lampertico L, Rapisarda F, Valsecchi L (2012). Formal and informal help-seeking for mental health problems. A survey of preferences of italian students. Clin Pract Epidemiol Ment Health.

[47] Rickwood DJ, Mazzer KR, Telford NR (2015). Social influences on seeking help from mental health services, in-person and online, during adolescence and young adulthood. BMC Psychiatry.

[48] Leung L (2007). Stressful life events, motives for Internet use, and social support among digital kids. Cyberpsychol Behav.

[49] Yip P, Chan WL, Cheng Q, Chow S, Hsu SM, Law YW, Lo B, Ngai K, Wong KY, Xiong C, Yeung TK (2020). A 24-hour online youth emotional support: Opportunities and challenges. Lancet Reg Health West Pac.

[50] Kingsbury M, Reme B, Skogen J, Sivertsen B, Øverland S, Cantor N, Hysing M, Petrie K, Colman I (2021). Differential associations between types of social media use and university students' non-suicidal self-injury and suicidal behavior. Computers in Human Behavior.

[51] Vannucci A, McCauley Ohannessian C (2019). Social Media Use Subgroups Differentially Predict Psychosocial Well-Being During Early Adolescence. J Youth Adolesc.

[52] Braun V, Clarke V (2020). Can I use TA? Should I use TA? Should I *not* use TA? Comparing reflexive thematic analysis and other pattern‐based qualitative analytic approaches. Couns Psychother Res.

[53] Braun V, Clarke V (2020). One size fits all? What counts as quality practice in (reflexive) thematic analysis?. Qualitative Research in Psychology.

[54] Braun V, Clarke V (2019). Reflecting on reflexive thematic analysis. Qualitative Research in Sport, Exercise and Health.

[55] (2019). Student Enrolment Statistics, 2019/20 (Kindergartens, Primary and Secondary Schools). Hong Kong Education Bureau, HKSAR.

[56] Siu AMH (2019). Self-Harm and Suicide Among Children and Adolescents in Hong Kong: A Review of Prevalence, Risk Factors, and Prevention Strategies. J Adolesc Health.

[57] Tabachnick BG, Fidell LS, Ullman JB (2007). Using Multivariate Statistics (5th edition).

[58] Kwon S, Park E, Kim K (2019). What drives successful social networking services? A comparative analysis of user acceptance of Facebook and Twitter. The Social Science Journal.

[59] Sun B, Mao H, Yin C (2020). Male and Female Users' Differences in Online Technology Community Based on Text Mining. Front Psychol.

[60] Balt E, Mérelle Saskia, van Bergen D, Gilissen R, van der Post P, Looijmans M, Creemers D, Rasing S, Mulder W, van Domburgh L, Popma A (2021). Gender differences in suicide-related communication of young suicide victims. PLoS One.

[61] AlFaris E, Irfan F, Ponnamperuma G, Jamal A, Van der Vleuten C, Al Maflehi N, Al-Qeas S, Alenezi A, Alrowaished M, Alsalman R, Ahmed AMA (2018). The pattern of social media use and its association with academic performance among medical students. Med Teach.

[62] Giunchiglia F, Zeni M, Gobbi E, Bignotti E, Bison I (2018). Mobile social media usage and academic performance. Computers in Human Behavior.

[63] Nesi J, Burke TA, Bettis AH, Kudinova AY, Thompson EC, MacPherson HA, Fox KA, Lawrence HR, Thomas SA, Wolff JC, Altemus MK, Soriano S, Liu RT (2021). Social media use and self-injurious thoughts and behaviors: A systematic review and meta-analysis. Clin Psychol Rev.

[64] Thorisdottir IE, Sigurvinsdottir R, Asgeirsdottir BB, Allegrante JP, Sigfusdottir ID (2019). Active and Passive Social Media Use and Symptoms of Anxiety and Depressed Mood Among Icelandic Adolescents. Cyberpsychol Behav Soc Netw.

[65] Verduyn P, Lee DS, Park J, Shablack H, Orvell A, Bayer J, Ybarra O, Jonides J, Kross E (2015). Passive Facebook usage undermines affective well-being: Experimental and longitudinal evidence. J Exp Psychol Gen.

[66] Phu B, Gow A (2019). Facebook use and its association with subjective happiness and loneliness. Computers in Human Behavior.

[67] Ye S, Ho K, Zerbe A (2021). The effects of social media usage on loneliness and well-being: analysing friendship connections of Facebook, Twitter and Instagram. IDD.

[68] Harris KM, Starcevic V, Ma J, Zhang W, Aboujaoude E (2017). Suicidality, psychopathology, and the internet: Online time vs. online behaviors. Psychiatry Res.

[69] Best P, Manktelow R, Taylor B (2014). Online communication, social media and adolescent wellbeing: A systematic narrative review. Children and Youth Services Review.

[70] Mar M, Neilson E, Torchalla I, Werker G, Laing A, Krausz M (2014). Exploring e-Mental Health Preferences of Generation Y. Journal of Technology in Human Services.

[71] Gandhi A, Luyckx K, Goossens L, Maitra S, Claes L (2018). Association between Non-Suicidal Self-Injury, Parents and Peers Related Loneliness, and Attitude Towards Aloneness in Flemish Adolescents: An Empirical Note. Psychol Belg.

[72] Chan M, Li TMH, Law YW, Wong PWC, Chau M, Cheng C, Fu KW, Bacon-Shone J, Cheng QE, Yip PSF (2017). Engagement of vulnerable youths using internet platforms. PLoS One.

[73] Utz S, Breuer J (2017). The Relationship Between Use of Social Network Sites, Online Social Support, and Well-Being: Results From a Six-Wave Longitudinal Study. J Media Psychol.

[74] Aguirre Velasco A, Cruz ISS, Billings J, Jimenez M, Rowe S (2020). What are the barriers, facilitators and interventions targeting help-seeking behaviours for common mental health problems in adolescents? A systematic review. BMC Psychiatry.

[75] Mesch G (2009). Social context and communication channels choice among adolescents. Computers in Human Behavior.

[76] Robinson J, Bailey E, Hetrick S, Paix S, O'Donnell M, Cox G, Ftanou M, Skehan J (2017). Developing Social Media-Based Suicide Prevention Messages in Partnership With Young People: Exploratory Study. JMIR Ment Health.

[77] Pourmand A, Roberson J, Caggiula A, Monsalve N, Rahimi M, Torres-Llenza V (2019). Social Media and Suicide: A Review of Technology-Based Epidemiology and Risk Assessment. Telemed J E Health.

[78] Renninger B (2014). “Where I can be myself … where I can speak my mind” : Networked counterpublics in a polymedia environment. New Media & Society.

[79] Stutzman F, Gross R, Acquisti A (2013). Silent Listeners: The Evolution of Privacy and Disclosure on Facebook. JPC.

[80] Livingstone S (2008). Taking risky opportunities in youthful content creation: teenagers' use of social networking sites for intimacy, privacy and self-expression. New Media & Society.

[81] Brown R, Fischer T, Goldwich D, Plener P (2020). "I just finally wanted to belong somewhere"-Qualitative Analysis of Experiences With Posting Pictures of Self-Injury on Instagram. Front Psychiatry.

[82] Curtis C (2010). Youth perceptions of suicide and help-seeking: ‘They'd think I was weak or “mental”’. Journal of Youth Studies.

